# Improving teamwork and communication in the operating room by introducing the theatre cap challenge

**DOI:** 10.1177/17504589211046723

**Published:** 2022-01-10

**Authors:** Anne Sophie HM van Dalen, Jan A Swinkels, Stan Coolen, Robert Hackett, Marlies P Schijven

**Affiliations:** 1 Department of Surgery, Amsterdam Gastroenterology and Metabolism, Amsterdam UMC, University of Amsterdam, Amsterdam, The Netherlands; 2 Department of Psychiatry, Amsterdam UMC, University of Amsterdam, Amsterdam, The Netherlands; 3 Department of Surgery, Amsterdam UMC, University of Amsterdam, Amsterdam, The Netherlands; 4 Department of Anesthesiology, 2205 Royal Prince Alfred Hospital, 2205 Royal Prince Alfred Hospital, Sydney, Australia

**Keywords:** Operating room, Surgical safety, Quality improvement, Teamwork, Surgical Safety Checklist, Closed-loop communication, Name stickers

## Abstract

**Objective:**

One of the steps of the Surgical Safety Checklist is for the team members to introduce themselves. The objective of this study was to implement a tool to help remember and use each other’s names and roles in the operating theatre.

**Methods:**

This study was part of a pilot study in which a video and medical data recorder was implemented in one operating theatre and used as a tool for postoperative multidisciplinary debriefings. During these debriefings, name recall was evaluated. Following the implementation of the medical data recorder, this study was started by introducing the theatre cap challenge, meaning the use of name (including role) stickers on the surgical cap in the operating theatre.

**Findings:**

In total, 41% (n = 40 out of 98) of the operating theatre members were able to recall all the names of their team at the team briefings. On average 44.8% (n = 103) was wearing the name sticker.

**Conclusions:**

The time-out stage of the Surgical Safety Checklist might be inadequate for correctly remembering and using your operating theatre team members’ names. For this, the theatre cap challenge may help.

**Provenance and Peer review:** Unsolicited contribution; Peer reviewed; Accepted for publication 30 August 2021.

## Introduction

The importance of clear communication in the operating theatre (OT) has been widely recognised (Espin et al 2020). Yet, ineffective communication is a major root cause of surgical adverse outcomes (Leonard et al 2004, Wahr et al 2013). The crew resource management principles, adapted from the aviation industry, emphasise the importance of using the closed-loop communication (CLC) technique in preventing adverse events (El-Shafy et al 2018). CLC includes three components: (1) an initial message that starts with stating the name of the recipient, known as *directed call out*, (2) verification by the named recipient, including repeating the critical aspect of the message, known as *check back* and (3) verification by the message sender that the recipient has interpreted the sent message correctly, known as *closing the loop* (Davis et al 2017, El-Shafy et al 2018). Accordingly, the World Health Organization (WHO) Surgical Safety Checklist (SSC) briefing includes an introduction stating name and role of all team members before start of a procedure. However, there is little data to support how name and role introductions improve name recall amongst staff (Birnbach et al 2017, Burton et al 2018). Simple strategies to remember and use each other’s names and roles, besides the SSC introduction round, writing down the names on a whiteboard and team briefing exist. In addition, the Patient Safety Network’s ‘Theatre Cap Challenge’ emphasises the importance of visible staff identification, by putting your name and role on your surgical cap when working in highly stressful environments such as the OT (Burton et al 2018). Some departments, such as the trauma room, already used name stickers to identify the staff, so this method may be easily rolled out in the OT (El-Shafy et al 2018).

During the roll out of the use of the theatre cap challenge at our medical centre, the aims of this study were to (1) evaluate if name and role instructions as part of the WHO’s SSC were actually completed, (2) how well team members were able to remember and recall each other’s name, and (3) evaluate the introduction of the theatre cap challenge.

## Methods

This study was part of a pilot study aiming to implement a video and medical data recorder (MDR) in an OT, used as a tool for structured postoperative multidisciplinary debriefings to improve surgical safety (van Dalen et al 2020b). Thirty-four laparoscopic (gastro-intestinal) procedures were recorded with the MDR and debriefed with the entire OT team outside the OT (in a neutral environment), using the MDR outcome report (van Dalen et al 2020a). The Works Council (staff representation) and Hospital Directorate approved the study. All subjects gave their written informed consent for participation in the MDR procedures and the MDR debriefings.

During the multidisciplinary debriefings of the MDR pilot study, the study coordinators hypothesised that the OT team members were often not able to remember the names of their peers and that miscommunication was one of the main topics during these debriefings (van Dalen et al 2020b).

**Figure 1:**
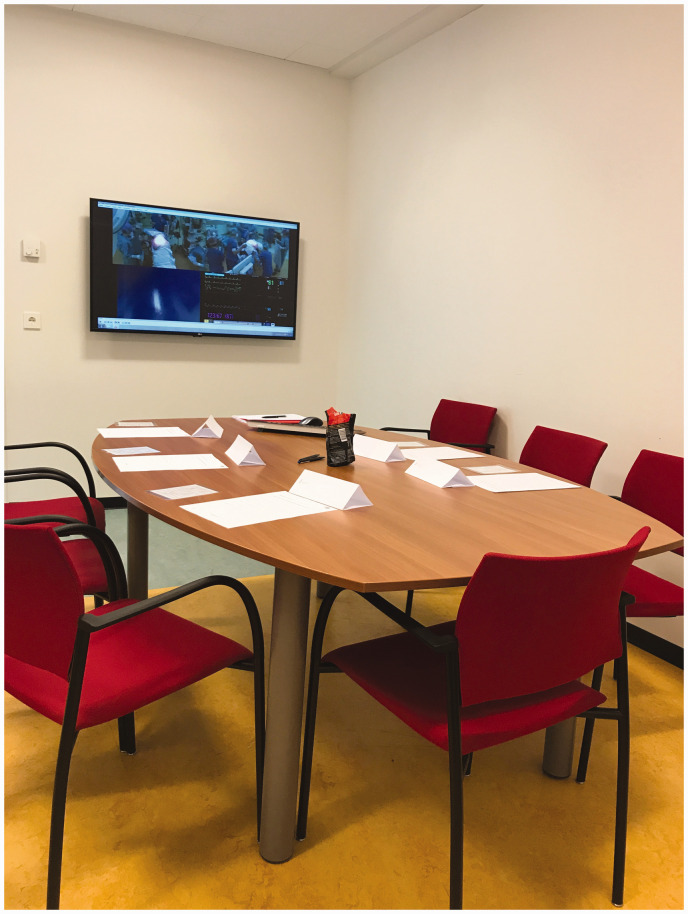
Name plates during the postoperative team debriefing sessions

Name recall was therefore evaluated by asking the participants (ie: the entire OT team), before the start of each postoperative multidisciplinary MDR debriefing, to write down the names of all the participating team members with whom they had worked during the particular case. Sitting at a table, they noted their team members’ names and pairing role on a paper sheet. The completed sheets were returned to the study coordinator. Subsequently, their own name was written on a triangular name plate, so that all names were visible throughout the debriefing (see Figure 1). Moreover, the study coordinator was present in the OT during all 34 recorded procedures and noted whether or not an official introduction round was carried out with the entire team in the OT, according to the SSC (Surgical Patient Safety System – SURPASS) (de Vries et al 2008, WHO 2009).

**Figure 2:**
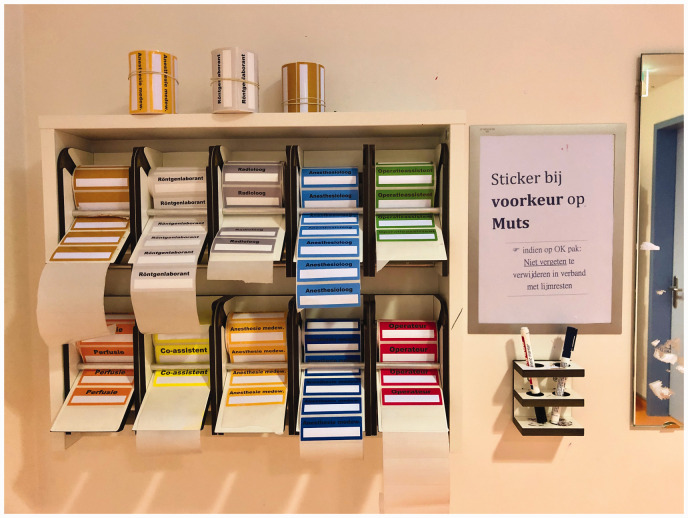
Name sticker station on the operating room complex, with a sign (on the right) kindly asking to put the stickers on the surgical cap

Following the results of the name recall evaluation, the theatre cap challenge was introduced by placing name and role sticker stations in the dressing rooms of the operating complex (November 2018), as shown in Figure 2. The OT staff was notified accordingly via email and asked to wear the name stickers on their surgical caps. Use of the name stickers was voluntary. Board members and team leaders acted as role models in wearing the name stickers.

## Results

The study coordinator observed that one out of four staff surgeons carried out an official introduction round, during which all team members present publicly said their full names including role. The SUPRASS item ‘confirm all team members have introduced themselves by name and role’ was in all 34 cases checked off as completed. All names and roles were noted on the whiteboard in the OT before start of procedure, usually by the circulating nurse, although surgeons also did write down their name with their phone number themselves.

In total, 238 postoperative questionnaires were completed directly after the 34 recorded surgical procedures. According to the specific OT team member filling out the questionnaire, in 82.4% (n = 196) of the cases it was stated that the entire OT team was indeed introduced.

In total, 41% (n = 40, out of 98) of the OT team members were able to recall all the names of their team members attending the postoperative MDR team debriefing. As shown in Table 1, the name of the primary surgeon was remembered most often (93%, n = 68) and the name of the medical intern least often (32%, n = 18). The primary surgeon could remember the name of the anaesthesiologist only on 50% of occasions (n = 14) and the scrub nurse’s name 58% of the time (n = 12). The anaesthesiologist could remember the name of the primary surgeon 75% of the time (n = 9) and the scrub nurse’s name 38% of the time (n = 8).

**Table 1:** Number of times the team member remembered the name of their fellow team member

	Did remember/know the name of:							
	Primary surgeon (N = 73)	Assisting surgeon (N = 55)	Anaesthesiologist (N = 49)	Anaesthesiology nurse (N = 49)	Scrub nurse (N = 53)	Circulating nurse (N = 44)	Medical intern (N = 57)	
Total times their name was remembered	93% (n = 68)	80% (n = 44)	59% (n = 29)	76% (n = 37)	64% (n = 34)	66% (n = 29)	32% (n = 18)	
Role in the OR								*P*-value^a^
Primary surgeon		14 (N = 14, 100%)	6 (N = 12, 50%)	11 (N = 13, 85%)	7 (N = 12, 58%)	8 (N = 12, 67%)	3 (N = 11, 27%)	*P* < 0.0001
Assisting surgeon	14 (N = 14, 100%)		6 (N = 7, 86%)	6 (N = 10, 60%)	6 (N = 9, 67%)	3 (N = 6, 50%)	4 (N = 8, 50%)	*P* < 0.0001
Anaesthesiologist	9 (N = 11, 75%)	6 (N = 8, 75%)		7 (N = 8, 88%)	3 (N = 8, 38%)	3 (N = 6, 50%)	0 (N = 6, 0%)	*P* < 0.0001
Anaesthesiology nurse	12 (N = 14, 86%)	9 (N = 10, 90%)	6 (N = 8, 75%)		6 (N = 8, 75%)	6 (N = 6, 100%)	3 (N = 6, 50%)	*P* < 0.0001
Scrub nurse	11 (N = 12, 92%)	5 (N = 9, 56%)	7 (N = 9, 29%)	5 (N = 7, 71%)		8 (N = 8, 100%)	3 (N = 9, 33%)	*P* < 0.0001
Circulating nurse	12 (N = 12, 100%)	3 (N = 6, 50%)	2 (N = 7, 29%)	5 (N = 5, 100%)	8 (N = 100%)		4 (N = 7, 57%)	*P* < 0.0001
Medical intern	10 (N = 10, 100%)	7 (N = 8, 88%)	2 (N = 6, 33%)	3 (N = 6, 50%)	4 (N = 50%)	1 (N = 6, 17%)		*P* < 0.0001

^a^Chi-square test.

As shown in Table 2, there was no significant difference between the times the OT team was introduced prior to the start of the surgical procedure, according to the questionnaire, versus times the names of the specific OT team members were remembered at the postoperative MDR team debriefing. There was no significant correlation between name and role introduction actually being performed and the percentage of correct name recall (*P*-value = 0.310, 96%CI –0.83 to 4.06).

**Table 2:** Number of times the team was introduced preoperatively versus times the names were remembered at the team debriefing

	‘Yes, names were introduced preoperatively’	‘No, names were not introduced preoperatively’	
Yes, name was remembered of:			*P*-value^a^
Primary surgeon (N = 73)	59 (94%)	9 (90%)	*P* = 0.67
Assisting surgeon (N = 55)	37 (80%)	8 (80%)	*P* = 1.0
Anaesthesiologist (N = 49)	25 (56%)	4 (100%)	*P* = 0.08
Anaesthesiology nurse (N = 49)	31 (76%)	6 (75%)	*P* = 0.97
Scrub nurse (N = 53)	30 (65%)	4 (57%)	*P* = 0.68
Circulating nurse (N = 44)	23 (61%)	6 (100%)	*P* = 0.58
Medical intern (N = 48)	16 (38%)	2 (33%)	*P* = 0.82

^a^Chi-square test.

About one year after implementation (September 2019), the theatre cap challenge was evaluated by asking a medical student, unfamiliar to OT staff, to count (on two randomly chosen mornings at the start of the working day and one time during the lunchtime break, for 1.5h) how many individuals, and who were actually wearing the name stickers. On average 44.8% (N = 230) was wearing the stickers whilst working in the OT. In 40.8% (N = 42), they had put them on the surgical cap and in 59.2% (N = 61) on the chest or name badge. Out of the 103 identified subjects in the theatre complex, 17 (16.5%) were surgeons, 29 (28.2%) were OT theatre nurses, 31 (30.1%) were anaesthesia nurses and 15 (14.6%) were medical interns.

We found that on average almost half of the OT staff (44.8%, n = 103 out of 230 observations) was now wearing the stickers on their surgical cap whilst working in the OT complex. Of this randomly observed sample (N = 103), 17 (16.5%) were surgeons, 29 (28.2%) were OT nurses, 31 (30.1%) were anaesthesia nurses and 15 (14.6%) were medical interns.

Those who did not want to wear the name stickers commented ‘I am not new’, ‘we do not wear them in an OT where everybody already knows each other’ or ‘it feels like kindergarten’. However, those who did wear them commented ‘it looks silly, but it works’, ‘I feel more part of the team when I am certain that everybody is able to use my name’, ‘I have been working here for 30 years and still do not know everybody’s name’ and ‘it is useful, because especially during stressful situations names are forgotten’.

## Discussion

During the pilot study MDR debriefings, participants realised how difficult it apparently is to remember each other’s names. Moreover, participants indicated they felt ashamed or awkward for not knowing the names of their colleagues, with whom they had worked multiple times before. The importance of awareness and education in communication skills in a high-risk environment such as the OT may hence not be underestimated (Catchpole & Russ 2015, Rydenfalt et al 2013). Davis et al (2017) demonstrated directed communication was associated with an increased likelihood of receiving a proper answer and confirmation that the message was received. Increased incidence of check backs (ie, as part of the CLC technique) reduced the number of ineffective communication events, provided opportunities for clarification of safety-critical information, and enhanced the OT team’s shared mental model. They also emphasised that addressing each other by name before sending the message may avoid unnecessary miscommunication.

Perhaps not surprisingly in daily practice with many checklists to complete, the name introduction item was usually ‘checked off’ by the team, without actually officially have taken place. Team members may say that they had worked with the same team members before; ‘We know each other already’. Yet, 59% of the time, the staff could not recall all the names of the team members whom they had performed the surgical procedure with. Non-compliance with this step of the SSC has been demonstrated in other studies (Levy et al 2012, Rydenfalt et al 2013) and once again highlights the problem with checklists. Just ‘checking the box’, by having it secured in the patient file does not mean the check has actually been performed, questioning its true value (Catchpole & Russ 2015, Rydenfalt et al 2013).

Usually, OT staff uses the team brief and the time-out as part of the five steps to safer surgery, before the start of the surgical procedure to introduce their name and role (Russ et al 2015). This may be helpful, but not suffice to adequately remember all the names. In certain situations or phases of a procedure, with staff fully focusing on important tasks, it is notably difficult to recall names, because faces are behind surgical caps and masks. Especially now during the COVID-19 pandemic, protective clothing and respiratory masks make it even more difficult to recognise each other in the hospital. In addition to that, team members may not always be able to make eye contact whilst concentrating behind the surgical drape or looking at the laparoscopic monitor. All these factors may complicate interaction and communication. The team has to respond to stressful situations, such as performing surgery during the COVID-19 pandemic, by promoting trust and coherency among colleagues. In these situations, it is particularly important to use the directed call out and CLC techniques.

Other studies have shown that the name of the primary surgeon is often the easiest to remember (Birnbach et al 2017, Burton et al 2018). Moreover, surgeons may be more often annoyed by the official introduction by names but nurses are usually more grateful (Haynes et al 2009). This may explain why nurses wear the name stickers more often. Studies have demonstrated that good leaders are often characterised by remembering and using the names of the people they work with (Lussier & Achua 2015). Although some may not see or understand the power of something as simple as knowing and using one another’s name, it is generally known that people feel more appreciated and are happier to help if you call them by their name, enhancing coherency of the team.

Limitations of this study are the small sample size and the single-centre study design. It was not possible to correlate the use of the name stickers to the number of communication events during the surgical procedures. We did not take into account the number of times a new OT member (name) was introduced per team and per case, which may have caused a bias. Future studies are needed to evaluate the actual impact of putting your name on your surgical cap on the use of the CLC technique, name recall, and subsequently the incidence of ineffective communication in the OT. This is the aim of the follow-up project of this pilot study, by using the improved version of the MDR (Saver 2019, Surgical Safety Technologies). Regardless, it may be considered important that every professional working in the OT realises the importance of the CLC technique, for which all team members need to be able to know and use each other’s name.

There are many reasons why people find it difficult to remember the names of their team members during surgery. Regardless, it remains difficult to remember and use names, even when the names are introduced prior to the start of the procedure, are written on a whiteboard and when team members have worked with one another multiple times before. Therefore, implementation of name stickers in the OT is recommended as it may facilitate the CLC technique in a simple manner. For this, a culture change in the OT environment is needed, which takes time and commitment (Burton et al 2018, Vaughn et al 2018). Patience and role modelling by leaders showing the way with using the name stickers to improve communication is important, and may promote positive safety behaviour, such as work satisfaction, providing feedback or error reporting (Catchpole et al 2021, Wakefield et al 2010). The results from this study recommend all team members to participate and embrace the theatre cap challenge, to create an even more positive safety culture by improving communication in the OT.

*No competing interests declared*
